# Phenolic Compounds from Water-Ethanol Extracts of* Tetrapleura tetraptera* Produced in Cameroon, as Potential Protectors against* In Vivo* CCl_4_-Induced Liver Injuries

**DOI:** 10.1155/2019/5236851

**Published:** 2019-03-03

**Authors:** Peter William Kemewele Saague, Bruno Moukette Moukette, Jacques Romain Njimou, Prosper Cabral Nya Biapa, Francine Nzufo Tankeu, Vicky Joseline Ama Moor, Constant Anatole Pieme, Jeanne Yonkeu Ngogang

**Affiliations:** ^1^Laboratory of Biochemistry, Department of Biochemistry and Physiological Sciences, Faculty of Medicine and Biomedical Sciences, University of Yaoundé I, P.O. Box 1364, Yaoundé, Cameroon; ^2^Department of Inorganic Chemistry, Faculty of Science, University of Yaoundé I, P.O. Box 812, Yaoundé, Cameroon; ^3^Department of Biochemistry, Faculty of Science, University of Dschang, P.O. Box 67, Dschang, Cameroon

## Abstract

**Background:**

Liver diseases are a global health problem. Medicinal plants are being increasingly used to manage a wide variety of diseases including liver disorders. The aim of this study was to investigate the antioxidant properties and hepatoprotective activity of polyphenolic extract from the fruits of* Tetrapleura tetraptera (T. tetraptera)*.

**Results:**

The extract of* T. tetraptera* was administered at doses of 50 mg/kg and 100 mg/kg for 07* per os* to rats before the induction of hepatotoxicity with of 2 ml/kg of 1:1 (v/v) carbon tetrachloride (CCl_4_) and olive oil through intraperitoneal route. The* in vitro* antioxidant and radical scavenging properties of* T. tetraptera* were conducted by the FRAP method, the phosphomolybdate method, and the inhibition potential of DPPH, ABTS, OH, and NO radicals. The extraction yield of* T. tetraptera* was 19.35%. This extract contains polyphenols (273.48 mg CAE/g DM), flavonoids (5.2549 mg SE/g DM), and flavonols (1.615 mg SE/g DM). This extract showed* in vitro* antioxidant activity, an inhibitor power of various free radicals, and radical scavenging potential dose-dependent. The fifty-percent inhibitory concentration of the extract (IC_50_) for the studied radical varied from 28.16 to 136 *μ*g/L. In rats treated with the extract of* T. tetraptera*, in a dose-dependent manner, the levels of hepatotoxicity markers such as alanine aminotransferase (ALT), aspartate aminotransferase (AST), and alkaline phosphatase (ALP) significantly increased while the enzyme activities of superoxide dismutase (SOD), catalase (CAT), and the level of reduced glutathione (GHS) significantly increased compared to the control group.

**Conclusions:**

The extracts from the fruit of* T. tetraptera* demonstrate antioxidant activity and hepatoprotective effects.

## 1. Background

Liver diseases are a global health problem. They are classified as acute or chronic hepatitis (inflammatory liver diseases), hepatosis (noninflammatory conditions), and cirrhosis (degenerative disorder resulting in liver fibrosis). Unfortunately, treatments for liver diseases are controversial because conventional or synthetic drugs for the treatment of these diseases are insufficient and sometimes cause serious side effects [1].

Several reports have demonstrated that oxidative stress is a major factor in the aetiology of hepatic disorders [2]. Oxygen reactive species (ROS) have been shown to damage biomolecules such as lipid and proteins at the cellular level leading to organ dysfunctions [2]. The antioxidant defence mechanisms are disturbed by oxidative reactive species. The increase in MDA levels, which is one of the end products of lipid peroxidation in the liver, and the reduction of hepatic GSH levels are important indicators in CCl_4_-intoxicated rats. Therefore, the potential hepatoprotective mechanism of action of this extract could be their inhibition of the oxidative radical of CCl_4_ or the protection of their cellular targets [3].

The prevention of the liver alteration is a critical current research issue, as several researchers have demonstrated protective activities of numerous compounds against prohepatotoxic agents [4, 5]. Natural products with antioxidant potential have been studied in this perspective. Antioxidant compounds from natural products have significant inhibitory effects on the deleterious activities of prohepatotoxins both* in vitro* and* in vivo* [5]. The mechanism involved in this effect includes the scavenging of free radical released by the xenobiotic or its activated form or the inhibition of the lipid peroxidation chain and/or the activation of antioxidant enzymes [6, 7].

*Tetrapleura tetraptera* (*T. tetraptera*) is a plant of the family of Mimosaceae mostly found in the south and west region of Cameroon. Its fruit has been used as a spice. It also serves as a medicinal product in the local folk medicine for the treatment of diabetes, intestinal worms, malaria, and fever [8, 9]. Our previous research demonstrated the free radical scavenging potential, antioxidant potential, and protective effects of an enzyme involved in oxidative stress of extracts from several parts of* T. tetraptera in vitro *[10, 11]. Furthermore, we demonstrated that the fruits and bark extracts from* T. tetraptera *contain a higher concentration of polyphenol mainly eugenol, quercetin, tyrosol, and catechin [11]. However, a study exploring the* in vivo* protective properties of the phenolic compounds from fruits of* T. tetraptera *might be essential in understanding its use against a variety of toxins. Therefore, this study has been carried out to investigate the* in vivo *hepatoprotective effect of this extract against the hepatic toxicity of CCl_4_ in rats.

## 2. Methods

### 2.1. Plant Material

The fresh fruits of* T. tetraptera* were harvested in the forest of the Mount Kala a small town near Yaoundé in the center region of Cameroon. The collected material was taken to the National Herbarium of Cameroon in Yaoundé where the samples were authenticated by botanists in comparison to the voucher specimens (N. 1858/ SRF).

### 2.2. Plant Sample Treatment and Extraction Process

**Figure 1:**
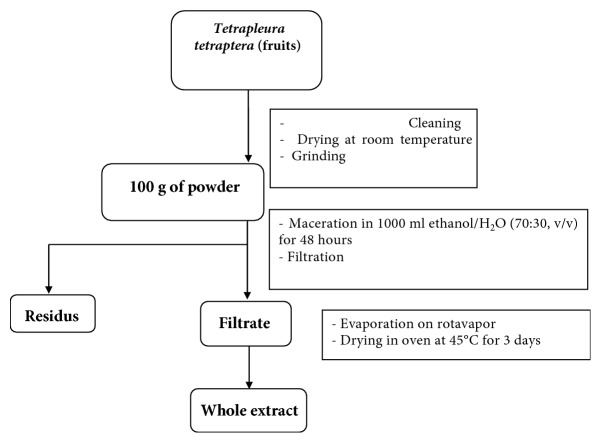
Extraction procedure of the crude extract.

The samples were dried at room temperature (27°C) and ground into powder. To obtain the extracts, the powder was soaked in a water/ethanol solution (30/70; pH=3) for 48h with the maceration ration of 1:10 (w/v). The filtration process was realized using a Buchner funnel and Whatman No° 3 filter paper. The solvent was removed by evaporation in an oven at 55°C. The dried extract was then collected (Figure 1) and kept for further experiments. The extract solution used for the experiments was prepared by diluting the extracts in water at the concentrations of 25, 50, 75, 100, 150, and 300 *μ*g/mL.

### 2.3. Animals

Adults male rats of* Wistar* strain weighing between 150 and 200 g were used for this experiments. The animals were kept in natural day-night cycle conditions and were fed ad libitum with standard laboratory diet and with tap water. The animals were allowed a one-week acclimatization period before the experiments. The protocol used in this study was in compliance with the guidelines of the committee of animal care and use of the University of Yaoundé I.

### 2.4. Experimental Design

The rats were divided into four groups of six animals. The first group received only distilled water administrated orally (1mL/Kg) per day during seven days and 2 mL/Kg of olive oil (1:1, v/v) intraperitoneally on the seventh day it served as the control group. The second group received only distilled water administrated orally (1mL/Kg) per day during seven days and 2 mL/Kg of CCl4 in olive oil (1:1, v/v) intraperitoneally on the seventh day. The third and four groups received the solution of extract administrated orally 50 mg/Kg and 100 mg/kg b.w., respectively, per day during seven days and 2 mL/Kg of CCl4 in olive oil (1:1 v/v) intraperitoneally on the seventh day. The rats were allowed two days before sacrificed on the ninth day by cervical decapitation under mild anesthesia. The blood was collected and centrifuged at 3000 rpm and the obtained serum was kept at -25°C for the biochemical assays while the livers were collected for histopathologic analysis.

### 2.5. *In Vitro* Free Radical Scavenging Assays

#### 2.5.1. Radical Scavenging Properties

*DPPH. *The DPPH inhibition assay was conducted according to previously described methods [12]. A volume of 3 mL of the plant extract was added to 1 mL of a solution of DPPH (0.1 mM in methanol). The mixture was vortexed and let to stay for 30 min away from the light. Then the optical density (OD) was read at 517 mn and the inhibition percentage was calculated as follows: *Savenging* *e*ff*ect* (%) = 100 × (*A*_*o*_ − *A*_*s*_)/*A*_*o*_, A_o_ = absorbance of the blank; A_s_ = absorbance of the sample.

*ABTS* ^*+*^. This assay was conducted following a standard method [13]. The ABTS radical were generated by the reaction of a solution of ABTS (7 mM) with potassium persulfate (2.45 mM). This solution was then diluted with ethanol to an OD of 0.70 ± 0.05 at 734 nm. This solution was kept in the dark room for 12 h. A volume of 25 *μ*L of the plant sample was added to 2 mL of the ABTS solution, and the OD was read after 6 min at 734 nm. The scavenging percentage was calculated as follows: *Savenging* *e*ff*ect* (%) = 100 × (*A*_*o*_ − *A*_*s*_)/*A*_*o*_, A_o_ = absorbance of the blank; A_s_ = absorbance of the sample.

*Nitric Oxide. *This assay is based on the reaction of Griess Illosvoy [14]. A volume of 1 mL of the extract solution was mixed with 0.5 mL of a SNP solution (Sodium nitroprusside (SNP) 10 mM in phosphate buffer (0.5 M, pH 7.4) and incubated for 1 h at 37°C. Thereafter, the solution was mixed with an equal volume of Griess solution (1% sulphanilamide in 2.5% phosphoric acid and 0.1% naphthyl ethylenediamine dihydrochloride in 2.5% phosphoric acid 1:1 (v/v)) [15]. The OD was read at 540 nm, and the inhibition percentage was calculated as follows: *Scavenging* *e*ff*ect* (%) = 100 × (*A*_*o*_ − *A*_*s*_)/*A*_*o*_, A_o_ = absorbance of the blank; A_s_ = absorbance of the sample.

*Hydroxyl (HO). *This assay was conducted using previously described methods [11]. In a volume of 1.5 mL of the sample, 60 *μ*L of Ferric chloride (1 mM), in 2.4 mL of 0.2 M phosphate buffer, pH 7.8, 90 *μ*L of 1,10-Phenanthroline (1 mM), and 150 *μ*L of H2O2 (0.17 M) were, respectively, added. The solution was vortexed and let to stand for 5 min. The OD was read at 560 nm, and the results were expressed in percentage using the following formula: *Scavenging* *e*ff*ect* (%) = 100 × (*A*_*o*_ − *A*_*s*_)/*A*_*o*_,  A_o_ = Absorbance of the blank; A_s_ = absorbance of the sample.

### 2.6. Total Antioxidant Properties* In Vitro*

#### 2.6.1. Ferric Reducing Antioxidant Power Assay (FRAP)

This assay is based on a standard method with slight modifications [16]. A volume of 75 *μ*L of the extract solution was added to 2 mL of the FRAP working solution (300 mM, pH 3.6). This solution was prepared by adding 50 mL of 2,4,6-Tri (2-pyridyl)-s-triazine (TPTZ) (10 mM) and 50 mL of ferric chloride (50 mM). The OD was read at 593, and the results were calculated using a standard curve of the vitamin C expressed as mg equivalent vitamin C/g of dried extract (mg eq VitC/g DE).

#### 2.6.2. Phosphomolybdenum Antioxidant Power (PAP)

This assay is based on a previously described method [17]. A volume of 10 *μ*L of the extract solution was added to 1mL of the PAP working solution (0.6 M sulphuric acid, 28 mM sodium phosphate, and 4 mM ammonium molybdate). The samples were mixed and incubated at 95°C for 90 min. The tubes were then let to cool down, and the OD was read at 695 nm. The results were expressed using the standard curve of the vitamin C and expressed as mg of ascorbic acid equivalent/g of dried extract (mg eq AS/g DE).

#### 2.6.3. Reducing Power

This assay was realized using the method described by Oyaizu (1996). A volume of 1 mL of extract was added to 2.5 mL of a phosphate buffer (0.2 M, pH 6.6) mixed with 2.5 mL of potassium ferrocyanide (1%). The mixture was vortexed and incubated at 50°C. A volume of 2.5 mL of trichloroacetic acid (10%) was then added; the tubes were centrifuged at 3000 rpm for 10 min. A volume of 2.5 mL of the upper layer was collected, mixed 0.5 mL of FeCl3 (0.1%), and diluted with 2.5 mL of distilled water. The OD was read at 700 nm.

### 2.7. Determination of the Phenolic Content of Extracts

#### 2.7.1. Total Phenolic Content

This assay was conducted following the method by the Folin–Ciocalteu method [18]. A volume of 200 *μ*L of the extract was added to 800 *μ*L of freshly prepared Folin Ciocalteu reagent and mixed with 2 ml of 7.5% sodium carbonate. Then the solution was diluted to 7 mL with deionized water and kept in the dark at ambient conditions for 2 h, and the OD was read at 765 nm. The results were expressed using the standard curve of the caffeic acid to express the results in mg caffeic acid/g dried extract (mg CA/g DE).

#### 2.7.2. Flavonoids

This assay was conducted using a standard method [19]. The sample (0.1 mL) was added to 0.3 mL distilled water and 0.03 mL of NaNO2 (5%). This solution was let to stand for 5 min at 25°C, and 0.03 mL of AlCl3 (10%) was added. After a further 5 min, the solution was mixed with 0.2 mL of 1 mM NaOH and diluted with 1 mL of distilled water. The OD was read at 510 nm. A standard curve of quercetin was used to express the results as (mg/g) dried extract (QE/g dried ext).

#### 2.7.3. Flavonols

This assay was conducted using previously described method [20]. A volume of 2 mL of the extract was added to 2 mL of an ethanolic solution of AlCl3 (2%), the solution was vortexed and 3 mL (50 g/L) sodium acetate solution was added and incubated for 2.5 h at 20°C. The OD was read at 440 nm. A standard curve of quercetin was used to express the results as (mg/g) dried extract (QE/g dried ext).

### 2.8. Determination of Oxidative Stress Makers

#### 2.8.1. Superoxide Dismutase (SOD) Activity

This assay was based on the inhibition of the autooxidation of epinephrine to adrenochrome. A volume of 150 *μ*L of the extract dilution was added to the epinephrine working solution [(3 x 10^−4^ M), Na_2_CO_3_ (10^−3^M), EDTA (10^−4^M)] and 350 *μ*mL of deionized water at a final volume of 1.5 mL. The OD was read at 480 nm at 0 sec for 180 sec. The SOD activity was expressed as Unit/min/mg of protein (UI/mg Prot.).

#### 2.8.2. Catalase Activity

This assay was conducted following previously described methods with modifications [21]. A volume of 0.5 ml of the serum was added to 1 mL of a phosphate buffer (pH 7.1; 50 mM), and the reaction was initiated by adding 0.1 mL of fresh 30 mM hydrogen peroxide. After 60 seconds, the reaction was 1.0 mL by adding a mixture of dichromate potassium (5%)/acetic acid (1:3v/v). The samples are then heated for 10 min in a Clifton water bath and allowed to cool and the OD read at 570 nm and the activity was expression which was similar to that of SOD (2.12.1.).

#### 2.8.3. Reduced Glutathione

This assay is based on the development of a yellow color of DTNB in the presence of sulfhydryl compounds [22]. A volume of 20 *μ*L of the serum is added to 3 mL of 5,5′-Dithiobis(2-nitrobenzoic acid) (DTNB) (0.01M); the mixture was then incubated for 1h, and the OD were read at 412 nm. The results were expressed as *μ*mole/L using the extinction coefficient *ε* =13600 /M.

#### 2.8.4. Total Protein Content

The level of protein in the serum was measured according to the protein kit supplier method (Randox kits N°: TP3869 ). This result served for the expression of the activities of the different enzymes per gram of protein.

### 2.9. Determination of Toxicity Markers

#### 2.9.1. Assay of Alkaline Phosphatase (ALP)

This test was conducted according to the kit supplier instructions (Randox kits N°: AP3877). A volume of 10 *μ*L of serum was mixed with 500 *μ*L of the reagent in a cuvette. The OD was read first at 405 nm and then every 30 seconds for 3 minutes.

#### 2.9.2. Assay of Alanine Transaminase (ALT) Activity

This test was conducted according to the kit supplier instructions (Randox kits N°: AL8006).

#### 2.9.3. Assay of Aspartate Transaminase (AST) Activity

It is the same method as that described for ALT (Randox kits N°: GOT1003). The difference was the ALT reagent that replaced the AST reagent.

### 2.10. Statistical Analysis

The software SPSS 20.0, IBM, USA, was used for our data analysis and XLstat version 7 (Addinsoft, USA) was utilized for the study of the correlations. The tests were realized in triplicate and the results represented as mean ± SEM. The data analysis was conducted using one-way analysis of variance (ANOVA) followed by a Tukey post hoc, and differences with p<0.05 were considered significant.

## 3. Results

### 3.1. Total Phenolic Content of* T. tetraptera*

The phenolic composition of the extracts from* T*.* tetraptera* fruit is represented in Table 1(a). The results showed that the total phenolic compound was 273.48 ± 1.82 mg CA/g DE while the concentration of flavonoid and flavonols was, respectively, 5.25 ± 0.04 QE/g dried ext. and 1.61 ±0.07 QE/g dried ext.

### 3.2. The Effect of* T. tetratptera *on the FRAP and the Phosphomolybdenum (PAP) Assays

The FRAP and the phosphomolybdenum antioxidant potentials of the extract of* T. Tetraptera* are showed in Table 1(b). These results showed that the concentration of ferric reducing the antioxidant power of* T*.* tetraptera* extract was 30.46 ± 0.26 mg eq VitC/g DE and that of PAP was 3.94 ± 0.24 mg eq AS/g DE.

### 3.3. The Effect of* T. tetratptera* on the Reductive Activity

The reductive activity of* T*.* tetraptera* extract pictured in Figure 3 showed that this activity was proportional to the concentration, from 1,81± 0.01 at the concentration of 25 *μ*g/mL to 1,87± 0.02 at the concentration of 300 *μ*g/mL. The vitamin C used as standard exhibited significantly (p<0.05) higher reductive activity at all the tested concentrations tested.

### 3.4. Scavenging Effects of* T. tetraptera* on Free Radicals (DPPH, OH, NO, and ABTS)

**Figure 2:**
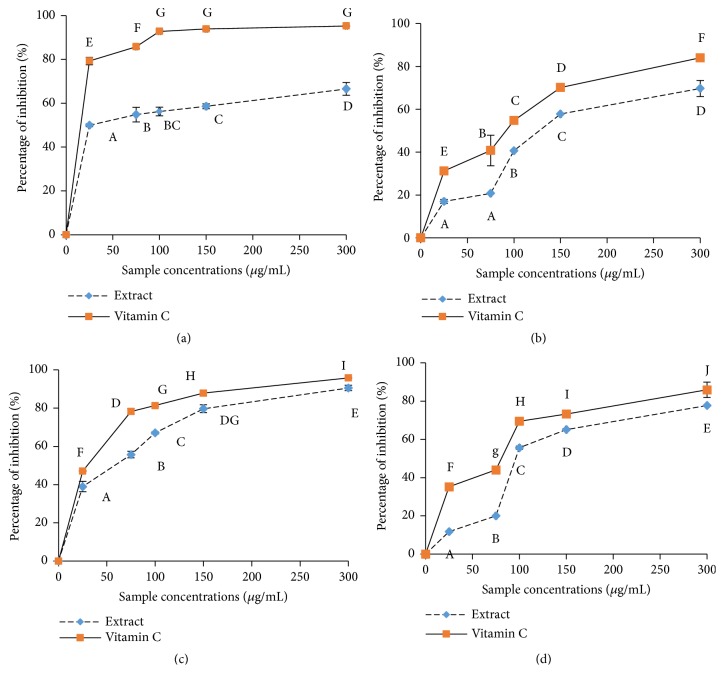
Study of the free radical scavenging effects the water-ethanol extract of* Tetrapleura tetraptera* ((a) DPPH, (b) ABTS, (c) OH, and (d) NO). Values are expressed as mean ± SD of three replicates. In the same concentration the values affected with different letter are significantly different at p<0.05. Extract: water-ethanol extract of* Tetrapleura tetraptera* fruits.

**Figure 3:**
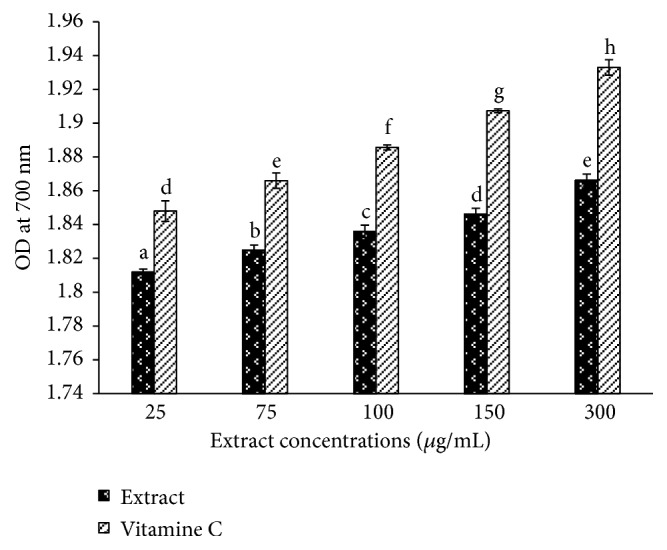
*Reductive power of the water-ethanol extract of Tetrapleura tetraptera.* Values are expressed as mean ± SD of three replicates. In the same concentration the values affected with different letter are significantly different at p<0.05. Extract: water-ethanol extract of* Tetrapleura tetraptera* fruits.

The scavenging effects of the* T*.* tetraptera* extract on the DPPH, OH, NO, and ABTS radicals are represented in Figure 2 and Table 2. This extract has shown a significant free radical scavenging potential characterized by an exponential increase of inhibitory percentage between the concentrations of 25 *μ*g/mL and 100 *μ*g/mL. The fifty-percent inhibitory concentration (IC50) of the extract varied from 28.16±3.17 to 137.67±2.08 *μ*g/mL depending on the type of radical (Table 2). The vitamin C used as standard showed the significantly (p<0.05) lowest IC50 value for the different radicals.

#### 3.4.1. In Vivo Antioxidant Potential of Extract of* T. tetratptera*

The effect of* T*.* tetratptera* extract on SOD, CAT activities, and GHS is represented in Table 3. The administration of CCl4 animals in the different groups has significantly (p<0.05) reduced the activities of SOD CAT and the level of GHS and MDA compared to the control. In this negative group (treated with only CCl4 2 mL/kg b.w) the value of the biochemical index was 29.38 ± 1.02 (10-4) for SOD, 15.09 ± 1.24 UI/mg Prot. for CAT, 2.97 ± 0.42 *μ*mol/l for GSH, and 123.09 ± 1.08 nM for MDA (Table 3). After oral administration of extract, we noted a significant (p<0.05) increase of all the antioxidant parameters tested in a dose-dependent manner. Compared to the negative control group, we noted that 1.71- to 3.86-fold augmentation of these parameters with CAT demonstrated the highest increment. However, the level of MDA significantly decreases 1.65-fold than the negative control.

#### 3.4.2. Effect of* T. tetratptera* on Toxicity Makers

Table 4 represents the effect of on the activity of ALAT, ASAT, and PAL. The results show that the treatment of the animals with CCl4 has increased significantly the concentration of toxicity markers from 30.33 ± 1.66 UI/L for ALP to 328.65 ± 19.63 UI/L for AST compare to the normal group. After administration of the extract, these enzymes significantly decrease in a dose-dependent manner. At 100 mg/kg, the activity of AST dropped significantly from 328.65 ± 19.63 to 146.65 ± 11.95 UI/L. However, none of these values was lower than those of the normal group.

#### 3.4.3. Effect* T. tetratptera* on the Liver Histology

**Figure 4:**
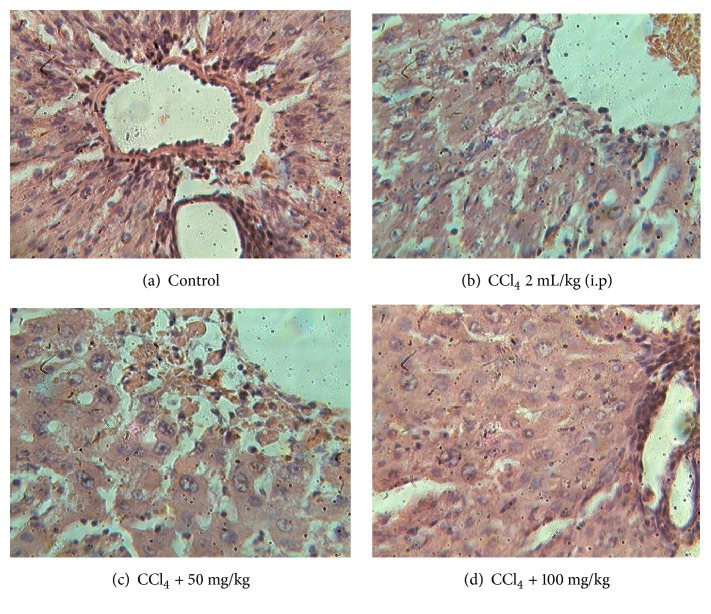
Microphotograph of rat liver. (a) Vehicle control group; (b) CCl_4_ 2 mL/kg (i.p) positive control group; (c) CCl_4_ + 50 mg/kg of extract group; (d) CCl_4_ + 1000 mg/kg of extract group (ip: intraperitoneal).

Figure 4 represents the pictures of liver histopathology. The histology of the liver of normal group of rats showed central vein surrounded by the hepatic cord of cells depicting obvious sinusoidal spaces delimited by endothelial cells (Figure 4(a)). In contrast, 48 h after the animals were treated with CCl4; the liver showed clear pale areas of necrosis and inflammatory cells around the central vein (Figure 4(b)). After treatment with the extract of* T*.* tetratptera*, these groups (Figures 4(b) and 4(c)) showed a decrease of the necrosed area and the level of inflammatory cells with maximal hepatoprotective at 100 mg/kg as compared to the untreated positive control.

## 4. Discussion

Polyphenolic compounds, widely distributed in plants, are known to have multiple biological effects such as anti-inflammatory, antidiabetic, antioxidant, and hepatoprotective [23, 24]. Recently, more attention has been given to plants which contain this class of compounds, with more emphasis on those used in the human diet [25, 26]. The fruit of* T. tetraptera* is a spice used as a condiment in Cameroon. The results from the current study revealed a high concentration of polyphenol and flavonoid in the extracts from the fruits. Our observation corroborates previous authors who demonstrated that the antiplasmodial effects of this plant might be correlated to his high phenolic content [11]. Polyphenols are a group of compounds in plants with high antioxidant potential. This antioxidant activity is mainly due to their redox potential that allows them to neutralize free radicals, singlet oxygen or decomposing peroxides [27]. The total antioxidant capacity of natural substances determined by the method of “FRAP” is based on the ability of these compounds to reduce TPTZ-Fe (III) complex into TPTZ-Fe (II) with an electron donation. Our results showed that water-ethanol extracts from the fruits of* T. tetraptera *have a high antioxidant potential (Table 1) as compared to other medicinal plants such as* Harungana madagascariensis *[21]. A positive correlation was also found between the total antioxidant power and the phenolic content of the extracts, as it augmented with increasing phenolic concentrations suggesting that these compounds could be responsible for this activity. Indeed, Bendary et* al.* (2013) demonstrated that the ability of a phenolic compound to act as electron donors was the critical factor of its antioxidant effect [28]. The study of the PAP potential of this extract was used as a complementary method to contribute demonstrating his antioxidant potential (Table 1). The result from the PAP test supported those obtained by the FRAP. The evaluation of the reducing capacity of these samples (Figure 1) brought more light on the mechanism involved in its antioxidant potential as we observed high reductive capacity in these extracts which was positively correlated to the PAP and FRAP. These activities could be explained by the higher concentration of flavonoids of* T. tetraptera*. Our previous HPLC analysis of the samples showed a high level in quercetin and eugenol, which contribute to an increase in the biological activity of medicinal plants [11].

Moreover, a positive and significant correlation has been demonstrated in previous work between the flavonoids level of plant extract and the PAP [11]. The extract of* T. tetraptera* also showed a free radical scavenging potential against DPPH, ABTS, OH, and NO radicals (Figure 2). The DPPH test is based on the discoloration of the DPPH radical in the presence of an electron donor compound [11]. The results showed that the extract from* T. tetraptera *scavenged both the DPPH and the ABTS radicals. The combined effects of the extract from* T. tetraptera *on these free radicals support the hypothesis that it has a high content in molecules capable of donating electrons and protons to free radicals, therefore, stabilizing them and avoiding macromolecule damage. These results strongly corroborate previous reports which showed the high ability of* T. tetraptera *to scavenge free radicals in a concentration-dependent manner and which were comparable to those of the ascorbic acid [9, 11]. Similar observations were made in the evaluation of the scavenging abilities against very toxic free radicals such as OH and NO. The results from these analyses corroborate previously published reports which showed the protective potential of phenolic compounds against oxidative mediated damage [29, 30]. The overproduction of reactive oxygen species promotes lipid peroxidation, protein oxidation and other cellular alterations which are considered to be essential mechanisms in the pathogenesis liver diseases [30]. Therefore, it is interesting to evaluate the in vivo biological properties of compounds capable of antioxidant activities. The liver provides many vital functions for the body, and it is mainly involved in xenobiotic detoxification. The liver is continuously exposed to the harmful action of drugs, chemicals, alcohol and other toxins which may lead to its damage [4]. Despite the advances in modern medicine, there are not enough effective drugs available for the protection of the liver against damage [30]. And, because of the side effects of conventional medications, people are becoming more interested in alternative and complementary medicines which tend to be safer [27, 31]. Mammalian cells possess effective defence mechanisms for radical detoxification which consist of a system of several antioxidant enzymes. The key enzymes of this system are superoxide dismutase (SOD), catalase (CAT), and peroxidases. SOD (EC 1.15.1.1) is an antioxidative enzyme which reacts against superoxide radicals and protects cells and tissues by converting the toxic superoxide radical anion (O_2_^–^) to hydrogen peroxide (H_2_O_2_) and O_2_. Its function depends explicitly on its quaternary structure. All changes in the environment may modify this structure and therefore the functionality of the SOD. In the present study, the extract from* T. tetraptera *markedly stimulated the increase in SOD activity in CCl_4_-induced liver-injury rats, indicating that the hepatoprotective effect of the extract may be associated with the reduction of superoxide level in the liver, which is consistent with the report by Ezeonu et* al.* (2017) who correlated the hepatoprotective effects of* Jatropha tanjorensis* to its flavonoid-rich methanolic fraction extract [32]. Catalases (EC 1.11.1.6) are ubiquitous heme enzymes that are found in aerobic organisms, ranging from bacteria to higher plants and animals. Functionally, catalases are related to peroxidases; both promote H_2_O_2_ oxidation by mechanisms that involve ferryl intermediates. Our results showed a substantial increase in the activity of catalase following the administration of the extract from* T. tetraptera.* This effect could contribute to the hepatoprotective properties of this sample as a reduction of H_2_O_2_ level in the body has been positively correlated to a decreased amount of cell injury [33]. Peroxidases (EC 1.11.1) are a large family of enzymes. These enzymes can convert hydrogen peroxide to water, obtaining the two hydrogen atoms it needs for this from a “donor” molecule. Our results showed a high peroxidase activity in the extract-supplemented groups, which is consistent with previous reports [32, 33]. The reduced glutathione levels were increased by treatment with* T. tetraptera*. Also, a significant negative correlation (p<0.05) had been observed between the SOD, catalase, and peroxidase activities and the level of polyphenol and flavonoids. Taken together, the results of the in vitro and in vivo antioxidant evaluation indicated that* T. tetraptera* showed good antioxidant activity, which may be attributable to its direct function in scavenging free radicals [32, 33]. The hepatoprotective properties of a diversity of plants have been previously reported, and these protective properties have been correlated with a high antioxidant potential [30]. Damage to the liver can be assessed through the determination the activity of enzymes such as alanine aminotransferase (ALT), aspartate aminotransferase (AST), and alkaline phosphatase (ALP). Indeed, their blood level is significantly increased after hepatic cytolysis [4]. In this study, an intraperitoneal injection of a single dose of CCl_4_ to the rats induced a significant increase of ASAT, ALAT, and PAL levels. Treatment of the rats with the extract from the fruits of* T. tetraptera* effectively protected the rats against CCl_4_-induced liver damage by reducing elevated serum ALT, AST, and PAL activities, as well as liver pathological changes. The result of the histological studies provided supportive evidence for the biochemical analysis. The histology of the untreated group showed hepatocyte cytoplasmic vacuolation which appeared to be consistent with glycogen and macrovesicular fat overload similar to previous author observations [32]. The tissue morphology of the group of rats treated with 100 mg/kg of the extract from the fruits of* T. tetraptera* showed a normal portal region with bile duct, veins, and arteries which are normal parts seen in liver architecture [31]. These results indicate that* T. tetraptera *could prevent CCl_4_-induced liver damage.

## 5. Conclusion

*T. tetraptera* extracts have demonstrated antioxidant activity and hepatoprotective properties. This plant can, therefore, be considered for more intense studies to isolate the active principle and describe its mechanism of action.

## Figures and Tables

**Table 1:** Phenolic composition and antioxidant power of the water-ethanol extract of *Tetrapleura tetraptera.*

*Assays*	*Extract*	*Polyphenols* *(mg CA/g DE)*	*Flavonoids* * (mg QE/g DE)*	*Flavonols* * (mg QE/g DE)*
Phenolic compounds	(100 *μ*g/mL)	273.48 ± 1.82	5.25 ± 0.04	1.61 ±0.07

Total antioxidant activity	(100 *μ*g/mL)		*Phosphomolybdate* * (mg eq AS/g DE)*	*FRAP* *(mg eq AS/g DE)*
			3.94 ± 0.24	30.46 ± 0.26

Values are expressed as mean ± SD of three replicates. Extract:water-ethanol extract of *Tetrapleura tetraptera* fruits

*mg CA/g DE*: mg equivalent of caffeic acid per g dried extract; *mg QE/g DE:* mg equivalent of quercetin per g of dried extract; *mg eq AS/g DE:* mg equivalent of ascorbic acid per g of dried extract

**Table 2:** Fifty percent inhibitory concentration value of the free radical scavenging potential of the water-ethanol extract of *Tetrapleura tetraptera.*

Samples	IC_50_ (*µ*g/mL)			
	DPPH	OH	NO	ABTS
Extract	28.16±3.17^a^	69.5±0.05^a^	111.67±1.15^a^	137.67±2.08^a^
Vitamin C	16.16±0.28^b^	54.33±0.28^b^	75.16±0.76^b^	88.33±5.68^b^

Values are expressed as mean ± SD of three replicates. In the same column, the values affected with different letter (a,b) are significantly different at p<0.05; Extract: water-ethanol extract of *T. tetraptera* fruits

**Table 3:** Effect of the water-ethanol extract of *Tetrapleura tetraptera* on some antioxidant markers *in vivo*.

	Serum concentration of antioxidant markers		
GROUPS	SOD	CAT	Glutathionne
	(10^−4^ nUI/mg of Prot.)	(UI/mg of Prot.)	(*μ*mol/l)
Control	35.86 ± 0.90^a^	87.50 ± 7.17^a^	5.15 ± 0.59^a^
CCl_4_ 2 mL/kg (i.p)	29.38 ± 1.02^b^	15.09 ± 1.24^b^	2.97 ± 0.42^b^
CCl_4_ + 50 mg/kg	44.05 ± 6.18^c^	36.87 ± 5.07^c^	4.29 ± 0.65a
CCl_4_ + 100 mg/kg	50.48 ± 2.75^d^	58.36 ± 8.03^d^	6.69 ± 0.68c

Values are expressed as mean ± SD of five replicates. In the same column, the values affected with different letter are significantly different at p<0.05; Extract:water-ethanol extract of *T. tetraptera* fruits

**Table 4:** Effect of the water-ethanol extract of *Tetrapleura tetraptera* on some toxicity markers *in vivo.*

	Toxicity Markers		
GROUPS	ALAT	ASAT	PAL
	(UI /L)	(UI/L)	(UI/L)
Control	20.57 ± 1.90^a^	77.98 ± 1.08^a^	12.69 ± 2.31^a^
CCl_4_ 2 mL/kg (i.p)	260.25 ± 21.44^b^	328.65 ± 19.63^b^	30.33 ± 1.66^b^
CCl_4_ + 50 mg/kg	166.96 ± 9.35^c^	182.63 ± 15.54^c^	26.42 ± 2.86^c^
CCl_4_ + 100 mg/kg	139.82 ± 19.69^d^	146.65 ± 11.95^d^	20.40 ± 2.22^d^

Values are expressed as mean ± SD of five replicates. In the same column, the values affected with different letter are significantly different at p<0.05; Extract:water-ethanol extract of *T. tetraptera* fruits

## Data Availability

The data used to support the findings of this study are available from Dr Constant Anatole Pieme upon request.

## Abbreviations

DPPH: 2,2-Diphenyl-1-picrylhydrazyl
FRAP: Ferric Reducing Ability of Plasma
Vit C: Vitamin C
ABTS: 2,2-Azinobis (3-ethylbenzthiazoline)-6-sulfonic acid
BHT: Butylated hydroxytoluene
RNS: Reactive nitrogen species
ROS: Reactive oxygen species
FRAP: Ferric reducing antioxidant power
TBA: Thiobarbituric acid
H_2_O_2_: Hydrogen peroxide
CAT: Catalase
CCl4: Carbon tetrachloride.
